# The Combination of Interferon-Alpha and Ponatinib Enables Faster and Deeper Molecular Responses in Patient with De Novo Blast Crisis of CML: Interferon-Alpha May Return as a CML Treatment

**DOI:** 10.1155/2021/5518727

**Published:** 2021-05-03

**Authors:** Kunio Hayashi, Kazuhiro Ikegame, Naoto Takahashi

**Affiliations:** ^1^Division of Hematology, Meiwa General Hospital, 4-31 Agenaruocho, Nishinomiya, Hyogo 663-8186, Japan; ^2^Division of Hematology, Hyogo College of Medicine, Mukogawacho, Nishinomiya, Hyogo 663-8501, Japan; ^3^Department of Hematology Nephrology and Rheumatology, Akita University, 1-1 Hondo 1-Chome, Akita 010-8543, Japan

## Abstract

In the era of tyrosine kinase inhibitor (TKI) treatment, its effectiveness in treating chronic myelogenous leukemia (CML) has been improved, ensuring the same prognosis as that of healthy people of the same age. However, there are some patients with de novo blast crisis that undergoes acute conversion from the time of diagnosis and does not respond to TKI treatment, especially in the older patients. Here, we present a case of an older patient with de novo lymphoid crisis who was first treated with a combination of TKI and chemotherapy, but it was difficult to maintain a durable deep molecular response (DMR). After he achieved major molecular response (MMR) or less, it was possible to suppress IS% to DMR by performing a combined treatment with interferon-*α* (IFN-*α*) and ponatinib. It is considered that DMR can be maintained by the combination of the two-way action of IFN-*α*, that is, the transfer of dormant CML stem cells to the cellcycle and the activation of a specific immune response to CML cells. This clinical result suggests the possibility of the re-emergence of IFN-*α*, which has been used a therapeutic drug in the past.

## 1. Introduction

Long-term survival in patients with chronic myelogenous leukemia (CML) was not possible to achieve until the introduction of tyrosine kinase inhibitor (TKI) therapy. Until the era of TKI treatment, allogeneic hematopoietic stem cell transplantation (allo-SCT) and interferon-*α* (IFN-*α*) therapy were the available treatment options; however, they were successful only in a limited number of people. TKIs have significantly improved survival outcomes in CML patients [1], and the possibility to discontinue TKI treatment for successful cases without recurrence has been recently explored [2, 3]. However, there are some CML patients who do not achieve a deep molecular response (DMR) and they have to continue TKI therapy in spite of various side effects. In the case of de novo blast crisis, it is much more difficult to achieve MRD [4]. Although allo-SCT can be selected as a treatment mode, it is associated with high risks and is generally not indicated for older patients. For patients with chronic phase CML (CML-CP) who cannot obtain optimal response, some investigators were using a combination of IFN-*α* and TKI for treatment [5, 6]. Through this combination treatment, they have succeeded in obtaining a durable DMR [7]. In this report, we present a case of a patient with de novo blast crisis treated with the same combination after the chemotherapy to obtain early DMR and maintain a stable condition. This combination of IFN-*α* and TKI suggests the possibility of a resurgence of IFN-*α* in the treatment of CML.

## 2. Case Presentation

**Figure 1:**
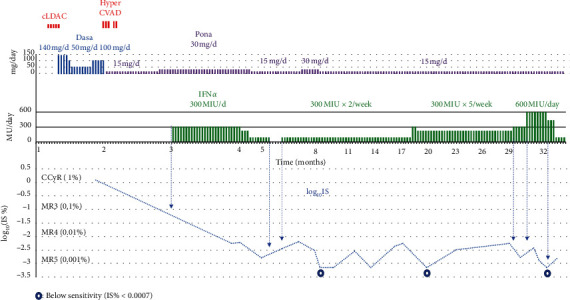
Clinical course of de novo blast crisis. Abbreviation: Nilo, nilotinib; Dasa, dasatinib; Pona, ponatinib; cLDAC, continuous infusion of low dose cytarabine (AraC); Hyper CVAD, hyperfractionated cyclophosphamide, vincristine, doxorubicin (adriacin), and dexamethasone.

A 68-year-old man was referred to our hospital, who presented with fever and loss of appetite. His routine annual health checkup results were normal and he had no history of an illness that required hospitalization. He had developed fever and cough approximately 10 days previously and had been treated by a doctor nearby. However, a blood test revealed a high white blood cell count of 25, 000/*μ*l and he was advised to visit our hospital. Laboratory test results on admission are shown in Table 1. Although he was suspected of having Philadelphia chromosome-positive acute lymphoid leukemia, the diagnosis of de novo lymphoid blast crisis CML was confirmed based on the results of the examinations. In the immunophenotypic data, blasts strongly expressed HLA-DR, CD34, CD19, CD10, and CD79a. The breakpoint was found in the major BCR, and the minor breakpoint region associated with Ph chromosome positive acute lymphoblastic leukemia was not detected. Dasatinib was initiated as the first-line therapy with a continuous low-dose cytarabine, but there was no hematological effect. The chemotherapy regimen was changed to the reduced doses of cyclophosphamide, vincristine, doxorubicin (adriacin), and dexamethasone (Hyper CVAD) with ponatinib 15 mg/day [6, 8]. Hematological remission was obtained on day 40 of this treatment. At that time, the bone marrow nucleated cell count was 1.1 × 10^4^/*μ*l and the proportion of blast cells was 1.4%, while the major BCR-ABL1 mRNA was 94 × 10^3^ copies/*μ*g. After the chemotherapy with Hyper CVAD, white blood cells were less than 1000/*μ*l for 20 days and neutrocytes were less than 500/*μ*l for 22 days during which time endogenous infection was complicated even under antibiotic treatment. In order to avoid worsening infectious diseases of febrile neutropenia, the chemotherapy was discontinued and treatment was changed to a combination of ponatinib 30 mg/day and IFN-*α* (300 MIU/day). In one month, the IS-PCR value was decreased to 0.0056% (MR4). The dose of ponatinib was reduced to 15 mg/day and IFN-*α* was continued at 300 MIU/day twice a week; after that, the IS-PCR value was decreased further to 0.0016%. When IFN-*α* was discontinued, the IS-PCR value was increased to 0.0064%. When IFN-*α* 300 MIU/day was restarted twice a week, the IS-PCR value was decreased to 0.0007% after 4.5 months and it fell below the detection sensitivity limit after another 4 months. The IS-PCR value was not stable and was increased again to 0.0055% after 3 months. When the dose of IFN-*α* was increased to 300 MIU/day five days a week, the IS-PCR value fell below the detection sensitivity in 2 months. Frequent measurements of the BCR-ABL1 transcript revealed fluctuations in its values, and IFN-*α* suppressed it and reduced the width of fluctuations in a dose-dependent manner. The course of treatment and the transition of IS% are shown in Figure 1. Because the de novo blast crises in older patients are maladapted for stem cell transplantation, such a deep molecular genetic response of BCR-ABL1 to INF-*α* is a great way to obtain a better prognosis.

## 3. Discussion

In the present case of the de novo lymphoid crisis in older patient, we succeeded to suppress the fluctuations in IS values and maintain DMR using the treatment of ponatinib combined with IFN-*α*. IFN-*α* therapy has become less commonly used with the advent of TKIs, but some studies suggested the possible effective use of IFN-*α*. In the Stop Imatinib (STIM) study, which first investigated the discontinuation of TKIs, having a history of IFN-*α* treatment was one of the prognostic factors that enhanced treatment-free response (TFR) [9]. The German CML and Scandinavian CML research groups have shown that a mode of combination treatment with IFN-*α* could elicit a faster and deeper response in high-risk CML patients than treatment with imatinib alone. These studies suggested that IFN-*α* provided clinical benefits [10].

TKIs have the effect of inactivating Ph-positive leukemia cells and inducing apoptosis but cannot eliminate these cells directly [3]. IFN-*α* acts on dormant leukemia stem cells, which cause the relapse, and recruits them into the cell cycle to be affected by TKI and further regulates gene expression in CML cells or induces cytotoxic T cells and natural killer cells [11]. Due to the synergistic effect of these various actions, the combined treatment of ponatinib and IFN-*α* is thought to suppress the progression of the disease. As observed in our case, the efficacy of IFN-*α* appears to be dose-dependent, but detailed studies to establish the optimal dosage and treatment duration of IFN-*α* are needed in the future.

## Figures and Tables

**Table 1:** Laboratory findings on admission.

*Peripheral blood*	
WBC	25, 200/*μ*l
Blast	78%
Stab	0%
Seg	1%
Lymph	20%
Mono	0%
Eo	1%
Baso	0%
RBC	220 × 10*e*4/*μ*l
Hb	7.1 g/dl
Ht	20.90%
Plt	2.2 × 10*e*4/*μ*l
NAP-R	89% (61∼99)
NAP-S	381 (170∼355)

*Coagulation*	
PT	13.3 sec
APTT	34.8 sec
Fibrinogen	321 mg/dl
FDP	3.5 *μ*g/*μ*l
D-dimer	1.2 *μ*g/ml
AT-III	110%

*Biochemistry*	
TP	7.0 g/dl
Alb	3.9 g/dl
Tbili	0.4 mg/dl
Dbili	0.1 mg/dl
AST	21 U/l
ALT	23 U/l
LDH	248 U/l
*γ*GTP	75 U/l
BUN	14.7 mg/dl
Cr	0.93 mg/dl
UA	5.4 mg/dl
eGFR	62.5 ml/min
Glu	71 mg/dl
CRP	1.8 mg/dl
Ferritin	1011.6 ng/ml

*Bone marrow*	
NCC	28.8 × 10*e*4/*μ*l
Mgk	+
Blast	98.80%
M/E	165

*G-Band karyotype of bone marrow*	
46, XY, t(9; 22) (q34; q11.2) [16]	
46, XY [4]	

*BCR-ABL1 transcripts*	
Major BCR-ABL1 18 × 10*e*5, HLA-DR+	

*Immunophenotype of blasts in bone marrow*	
CD34+, HLA = DR+, MPO−, CD3−, TdT+, CD19+, CD79a+, CD13±	

Abbreviation: WBC, white blood cells; blast, myeloblast; myelo, myelocytes; meta, metamyelocytes; stab, stab cells; seg, segmented cells; lym, lymphocytes; mono, monocytes; eo, eosinophiles; baso, basophiles; Hb, hemoglobin; Ht, hematocrit; Plt, platelet; NAP-rate, neutrophil alkaline phosphatase activities-rate; NAT-score, neutrophil alkaline phosphatase activities-score; NCC, nuclear cell counts; Mgk, megakaryocytes; M/E, myeloid erythroid ratio; PT, prothrombin time; APTT, activated partial thromboplastin time; FDP, fibrin/fibrinogen degradation product; AT-III, antithrombin-III.

## Data Availability

The authors confirm that the data supporting the findings of this study are available.
